# Etude séro-épidémiologique de la leishmaniose canine au centre du Maroc

**DOI:** 10.11604/pamj.2014.19.248.4563

**Published:** 2014-11-07

**Authors:** Hajiba Fellah, Oursula Doughmi, Saâd Maniar, Abdelhakim El Ouali Lalami

**Affiliations:** 1 Laboratoire de Référence des Leishmanioses, Institut National d'Hygiène, 27 Avenue Ibn Batouta, Agdal 11400 Rabat, Maroc; 2 Laboratoire Régional de Diagnostic Epidémiologique et d'Hygiène du Milieu, Direction Régionale de la Santé, Hôpital El-Ghassani, Fès, Maroc; 3 Observatoire Régional de la Santé, Direction Régionale de la Santé, Hôpital EL Ghassani, Fès, Maroc

**Keywords:** Leishmaniose viscérale, leishmaniose canine, sérologie, Fès, My Yacoub, Maroc, visceral leishmaniasis, canine leishmaniasis, serology, Fes, My Yacoub, Maroc

## Abstract

Dans le monde, la leishmaniose viscérale humaine est connue pour avoir comme principale source d'infection les Canidés domestiques et sauvages. Au centre du Maroc, les données épidémiologiques, cliniques et parasitologiques sur la leishmaniose canine, sont quasiment inexistantes. Ce travail traite une étude prospective au cours de laquelle 61 sérums canins ont été analysés par un test rapide et par l'immunofluorescence indirecte. La sensibilité du test rapide par rapport à celle de l'immunofluorescence indirecte (IFI) est de 33,33%. La fréquence de la maladie chez les chiens s’élève à 9,83% (Test Rapide) et 24,59% (IFI). 73,33% des cas canins positifs à la sérologie sont asymptomatiques. Ce sont les jeunes chiens de moins de 5 ans qui sont les plus fréquemment atteints avec une sensibilité de la race Berger Allmand à l'infection. Cette étude a permis de mettre en évidence la présence de chiens leishmaniens (15 chiens séropositifs parmi 61) et de prouver l'existence du réservoir canin. Une stratégie de prévention active doit être mise en place.

## Introduction

L'importance des leishmanioses dans le monde est illustrée par le nombre annuel de nouveaux cas qui se chiffre entre 1,5 à 2 millions [1]. En Afrique du Nord, trois espèces de Leishmania, associées à des caractéristiques environnementales, épidémiologiques et cliniques distinctes, sont responsables de la maladie, à savoir *Leishmania infantum*, *Leishmania*. major et *Leishmania*. tropica [2]. Au Maroc, la morbidité de la leishmaniose viscérale due à *Leishmania* (*L*.) *infantum* fluctue en moyenne autour de 152 cas par an [3]. Le centre du Maroc (Ville de Fès et ses alentours) est touché par les leishmanioses quelles soient cutanées ou viscérale [4]. Cette dernière est une anthropozoonose endémique des pays du bassin méditerranéen, d'Amérique du Sud et de la Chine [5], pour laquelle il semble que l'espèce canine est le réservoir du parasite et également la source indirecte d'infection de l'homme. La seule étude réalisée dans la région de Fès en 1997 traitait “les variations du taux d'anticorps anti *Leishmania infantum* chez une population canine” [6]. Depuis, la situation séro-épidémiologique de la Leishmaniose Canine reste méconnue.

## Méthodes

**Période et lieu de l’étude:** Cette étude a été réalisée en 2009 durant la saison printanière dans divers établissements à savoir: L'Américain Fondouk (Clinique vétérinaire) où ont été réalisés les prélèvements sur les chiens à propriétaire; La fourrière (Abattoir de Fès) où ont été effectués les prélèvements sur les chiens errants capturés avant d’être euthanasiés; L'unité de parasitologie du Laboratoire Régional du Diagnostic Epidémiologique et d'Hygiène du Milieu (LRDEHM) de la Direction Régionale de la Santé à Fès où a été réalisé le traitement des échantillons prélevés; Le Laboratoire de Référence des Leishmanioses de l'Institut National d'Hygiène (LRLINH) où a été accompli la confirmation et le sérotypage des échantillons prélevés.

**Echantillonnage canin et prélèvements:** Les chiens étudiés ont été divisés en deux groupes: Le premier groupe est composé de 52 chiens à propriétaires, venant en consultation à l'Américain Fondouk. Pour chaque chien, une fiche de renseignements a été soigneusement remplie à la lumière des données fournies par le propriétaire et l'examen clinique. Les renseignements recueillis concernaient l’âge, le sexe, la race et les signes cliniques. Il est à noter que l’âge des chiens a été retenu sur des éléments de l'interrogatoire et l’état de la dentition de l'animal. Le second groupe a été composé de 9 chiens errants, capturés par le personnel de la fourrière. Tous les chiens capturés ont été euthanasiés dans les 48h qui suivent la capture. Ces chiens présentaient des signes cliniques d'appel de la leishmaniose canine, dont nous citons la dermatite sèche, la dépilation et l'amaigrissement. Les prélèvements sanguins ont été réalisés sur l'ensemble des 61 chiens. Il s'agit de prélèvements de sang veineux sur tube sec. Les échantillons sanguins ont été directement acheminés dans une glacière à 4°C au LRDEHM pour leur traitement. Les sérums ont été recueillis dans des tubes eppendorf après centrifugations à 4000tr /4mn et conservés à -20°C.

**Test Rapide (TR):** Il s'agit du Test Rapide « OnSite leishmania IgG/IgM Combo Rapid test » de CTK Biotech. Chaque kit contient 30 dispositifs d′essai, chacun scellé dans un sachet en aluminium avec trois éléments à l′intérieur: un dispositif de cassette, un compte-gouttes en plastique et un déshydratant. Un diluant pour les échantillons (une bouteille de 5 mL) est aussi présent dans le kit ainsi qu'une notice. Le test rapide est une méthode immunochromatographique permettant la détection d'anticorps anti-leishmanies présents dans le sérum du chien. Le sérum a été décongelé à température ambiante. Le test rapide utilise une bandelette sensibilisée par l'antigène recombinant de *L. donovani*. 30 à 45µl du sérum ont été déposés dans le puits de la cassette et une même quantité de diluant lui a été ajoutée. La lecture a été effectuée 15 mn après la migration. Si le sérum contient des anticorps anti-leishmanies, ils se lieront à l'antigène recombinant présent dans le puits et lors de la migration, ils seront capturés par les anticorps anti IgG ou IgM. L'apparition d'une bande bordeaux couplée à une autre bande contrôle, témoigne que le sérum est positif. Un sérum négatif aura comme résultat l'apparition d'une seule bande contrôle.

**Immunofluorescence indirecte (IFI):** L'antigène utilisé est constitué par les formes promastigotes de la souche MO/PAR/LEI/07, préparée au LRLINH. Six dilutions (du 1/25 au 1/1600) de chacun des sérums à tester ont été effectuées. Après incubation à 37° C en chambre humide et lavage en tampon PBS (pH 7,2), un conjugué anti-IgG/A/M de Bio-Rad a été utilisé. Les lames confectionnées ont été observées au microscope à fluorescence. Le titre-seuil du 1/100 a été retenu.

**Traitement des données:** Nous avons utilisé le logiciel Excel pour traitement des données et pour la confection des graphiques.

## Résultats

### Etude prospective

**Figure 1:**
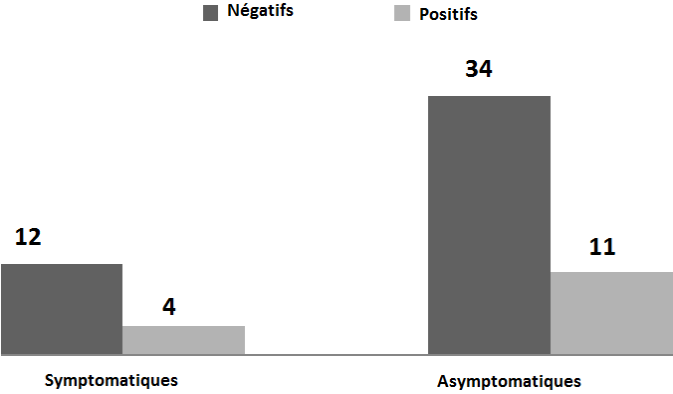
Répartition des chiens étudiés selon la symptomatologie

**Figure 2:**
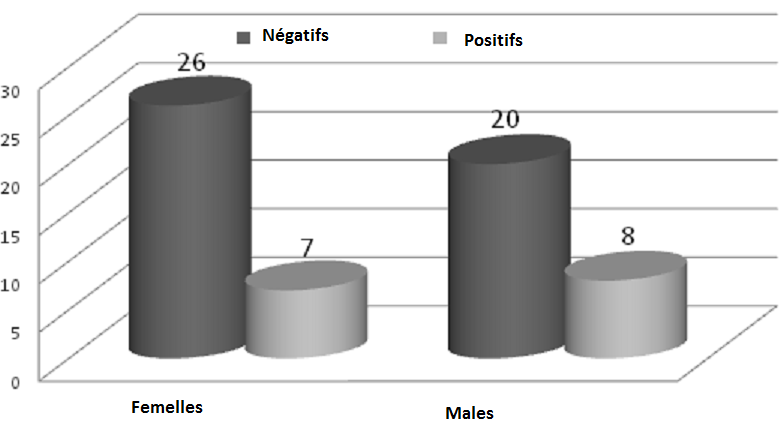
Répartition des chiens étudiés selon le sexe

**Figure 3:**
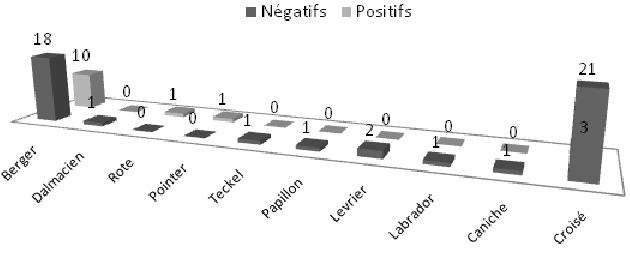
Répartition des chiens étudiés selon la race

**Figure 4:**
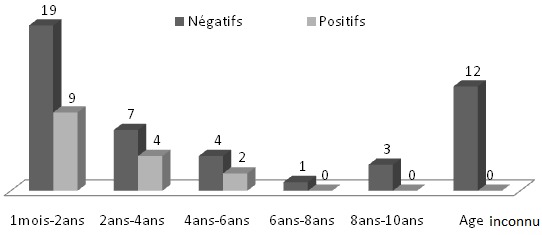
Répartition des chiens étudiés selon les tranches d’âge

Sur les 61 sérums de chiens contrôlés, 15 ont été positifs (soit 24,59%) au titre-seuil du 1/100 en IFI, et 6 (soit 9,83%) le sont également au TR. Les résultats de positivité du Test Rapide par rapport à l'IFI font apparaitre une faible sensibilité (vrais positifs/vrais positifs + faux négatifs) 33,33% et une bonne spécificité (vrais négatifs/vrais négatifs + faux positifs) 97,87%. L'analyse du graphique 1 montre que la majorité des cas positifs à la leishmaniose canine, soit 73,33% étaient asymptomatiques. Les chiens restants souffraient des symptômes suivants: dépilation, amaigrissement, abattement, dermatite, nodules multiples et difficulté à marcher (Figure 1). La population étudiée des chiens comprenait plus de femelles que de mâles avec un sex-ratio de 1,17. Le graphique 2 montre que l'infection ne dépond pas du sexe étant donné que le nombre de cas positifs femelles est presque égal à celui des mâles (Figure 2). La majorité des cas des chiens diagnostiqués étaient des Berger parmi lesquels 10 Bergers Allman, soit 66,66% étaient séropositifs à la leishmaniose canine et quatre d'entre eux étaient symptomatiques (Figure 3). Les autres races de chiens (Dalmatien, Rote, Pointer, Teckel, Papillon, Lévrier, Caniche et Labrador) ont été rarement rencontrées lors de notre étude et ne présentent quasi aucun cas de leishmaniose canine à part chez les Pointers et les Rotes où les seuls cas diagnostiqués se sont avérés positifs. Quant aux chiens croisés, nous avons réalisé 24 prélèvements parmi lesquels 3 sérums ont été identifiés positifs à la leishmaniose canine. La Figure 4 montre une répartition des chiens étudiés selon l’âge et selon l'atteinte par la leishmaniose canine. Nous avons remarqué que 45,9% des chiens étudiés étaient d’âge comprit entre quelques mois et 2 ans, parmi lesquels nous avons trouvé 9 cas de leishmaniose canine. Six autres cas positifs appartenaient à la tranche d’âge 2 ans-5 ans (Figure 4). D'après la Figure 4, nous en déduisons également que ce sont les jeunes chiens, d’âge inférieur à 5 ans, qui sont les plus sensibles à la maladie à cause de leurs faibles réactions immunitaires. Quant aux chiens errants dont la plupart sont suspects de rage, nous n'avons pas pu examiner leur dentition et de ce fait leurs âges restent indéterminés.

## Discussion

D'après les résultats de l'Immunofluorescence indirecte et du test rapide, les fréquences respectives de positivité par rapport aux animaux examinés (24,59% et 9,83%) ont été très supérieures aux valeurs habituellement retrouvées en zone d'enzootie leishmanienne [7]. Les résultats déduits du graphique 1 sont distincts de ceux trouvés par Harrat et al. en 2002 [8] à Algérois qui ont rapporté que parmi 666 cas positifs à la leishmaniose canine, 70% étaient symptomatiques dont les signes cliniques communs étaient l'amaigrissement et les lésions cutanées, 25% étaient des porteurs sains alors que 5% avaient un diagnostic non précis. L’étude réalisée par Aoun et al. en 2004 [9] en Tunisie a rapporté que 80% des cas positifs avaient une altération générale. Quant à l’étude réalisée à Nador par Rami et al. en 2003 [10], elle rapporte que 38,9% des chiens positifs étaient asymptomatiques. Les 12 cas symptomatiques qui se sont révélés négatifs à la leishmaniose canine, parmi lesquels nous citons les chiens errants dont le diagnostic clinique révélait de fortes probabilités de leishmaniose, peuvent être atteints d'autres affections liés au mode de vie sévère de ces animaux (manque de nourriture, maladies fréquentes’). D'autre part, Bouratbine et al. en 2005 [11], ont confirmé par la sérologie la maladie, chez un ensemble de 49 chiens symptomatiques (23 ruraux semi-errants et 26 citadins de races importées), à l'issue d'une étude sur les données épidémiologiques, cliniques et parasitologiques concernant la leishmaniose généralisée canine en Tunisie. Dans ce même pays, un travail similaire de Chargui et al., en 2007 [12], réalisé sur des sérums de 250 chiens a permis de confirmer l'augmentation de l'infection dans la région de Sfax et sa propagation vers le Sud de la Tunisie.

Au Brésil, une étude réalisée par Silva OA et al., en 2007 [13], sur un total de 503 sérums collectés au près de chiens asymptomatiques choisis arbitrairement sans critère d'aspect clinique, de race, de sexe ou d’âge et de provenance de 24 hameaux ruraux d'une municipalité, a mis en évidence également 62 cas positifs par la technique de l'IFI, soit 12,3%, avec 73% des positifs à un taux de 1,40. En 2012, Bessad A. et al. [14], ont rapporté l'isolement, pour la première fois, du parasite *L*. *infantum* chez un chacal doré (Canis aureus) capturé dans un village au nord de l'Algérie. Le résultat déduit de la Figure 2 concorde avec ceux des autres études réalisées au cours des dernières années, dont nous citons celle de Rami et al. en 2003 [10] à Nador, et celle de Aoun et al. en 2004 [9] en Tunisie. L’étude réalisée par Crotti et al. [15] en Italie en 2007, vient bousculer cette observation. Elle rapporte une prédilection sexuelle chez les mâles qui s'avèrent être plus sensibles à *Leishmania infantum*. D'après la Figure 3, nous pouvons déduire que les résultats obtenus au cours de notre étude concordent avec ceux trouvés à Algérois par Harrat et al. en 2002 [8] qui ont rapporté que 80% des séropositifs étaient des Bergers Allman, ainsi qu'avec les résultats trouvés en Italie par Crotti et al. en 2007 [15] qui ont trouvé une prévalence de la leishmaniose canine chez la même race.

Au Maroc, les Bergers Allmand sont souvent utilisés pour la surveillance des maisons et des parkings. La prévalence élevée de la leishmaniose chez cette race canine semble liée à son activité, en dehors des foyers, qui l'expose pendant la nuit aux piqûres de phlébotomes. Les résultats déduits de la Figure 4 sont discordants par rapport à ceux de Najjar et al. en 2000 [6] qui ont rapporté plus de cas positifs de leishmaniose canine chez des chiens adultes à la province de Séfrou et Zouagha My Yacoub. L’étude réalisée par Crotti en 2007 [15] a montré une distribution bimodale des cas séropositifs à la leishmaniose canine selon l’âge. Le premier pic est représenté par les chiens de moins de 3 ans, quant au second ce sont les chiens de 8 à 10 ans qui sont les plus atteints. Selon une autre étude réalisée en Tunisie sur la leishmaniose canine en 2005 par Bouratbine A. et al. [11], les chiens séropositifs sont plus jeunes (soit

Actuellement, nous pouvons affirmer que la présence de la leishmaniose canine dans cette région du centre du Maroc peut être liée à l'affection humaine et aux cas de la leishmaniose viscérale enregistrés par les services de la santé durant ces dernières années. En effet, la présence de vecteurs de la maladie dans cette région notamment *Ph. Longicuspus* et *Ph. Perniciosis* a été également confirmé par de nombreux auteurs [2, 16]. Ainsi, nous pourrons certifier la présence des trois éléments du cycle de *Leishmania infantum*, à savoir: Réservoir-Vecteur-Infection. En termes de santé publique, l’élimination des chiens infectés et l'abattage des chiens errants n'auront probablement que peu d'effets sur l'incidence de la maladie humaine [17, 13]. Il semble plus utile et primordiale de développer l’éducation, la sensibilisation et la communication pour la santé et d'inciter les malades notamment les cas porteurs de signes évocateurs de la pathologie de la leishmaniose viscérale à se rendre dans des établissements hospitaliers pour une prise en charge adéquate. Notre étude bien que préliminaire, a permis de mettre en évidence la présence de la leishmaniose canine dans la région de Fès comme c'est le cas dans d'autres zones du Maroc notamment au Nord [18, 19]. Cette situation doit inciter à plus de vigilance et de contrôle par des mesures de lutte intégrées et efficaces.

## Conclusion

Cette étude prospective nous a permis de mettre en évidence la présence de chiens leishmaniens (15 chiens séropositifs parmi 61) et de prouver l'existence du réservoir canin. D'autres études doivent être entamées pour d'une part évaluer par rapport à l'IFI et le TR, des techniques telles que la PCR ou la culture in vitro dans le diagnostic de l'infection par *Leishmania infantum* dans le sang des chiens et, d'autre part, d'estimer la prévalence de la leishmaniose canine au centre du Maroc.
